# Glycomimetic antagonists of BC2L-C lectin: insights from molecular dynamics simulations

**DOI:** 10.3389/fmolb.2023.1201630

**Published:** 2023-05-31

**Authors:** Giulia Antonini, Monica Civera, Kanhaya Lal, Sarah Mazzotta, Annabelle Varrot, Anna Bernardi, Laura Belvisi

**Affiliations:** ^1^ Università degli Studi di Milano, Dipartimento di Chimica, Milano, Italy; ^2^ Univ. Grenoble Alpes, CERMAV, CNRS, Grenoble, France

**Keywords:** lectins, glycomimetics, molecular dynamics simulations, C-fucosides, fucosyl amides

## Abstract

Opportunistic infections from multidrug-resistant pathogens such as *Burkholderia cenocepacia* are a threatening risk for hospital-bound patients suffering from immunocompromised conditions or cystic fibrosis. *B. cenocepacia* BC2L-C lectin has been linked to bacterial adhesion and biofilm formation, thus hindering its activity is seen as a promising strategy to reduce the severity of the infection. We recently described the first bifunctional ligands of the trimeric N-terminal domain of BC2L-C (BC2L-C–Nt), capable of simultaneously engaging its fucose-specific sugar binding site and a vicinal region at the interface between two monomers. Here, we report a computational workflow for the study of these glycomimetic bifunctional ligands in complex with BC2L-C-Nt, aimed at investigating the molecular basis of ligand binding and the dynamics of glycomimetic/lectin interactions. In particular, we evaluated the use of molecular docking in the protein trimer, followed by refinement using MM-GBSA re-scoring and MD simulations in explicit water. Computational results were compared to experimental data derived from X-ray crystallography and isothermal titration calorimetry. The computational protocol proved suitable to provide a reliable description of the interactions between the ligands and BC2L-C-Nt, highlighting the contribution of MD simulations in explicit solvent for a good fit with the experimental observations. The information achieved in the study and the whole workflow appear promising for the structure-based design of improved BC2L-C-Nt ligands as novel antimicrobials with antiadhesive properties.

## 1 Introduction

The opportunistic Gram-negative pathogen *Burkholderia cenocepacia* is a globally spread multidrug-resistant bacterium that represents a severe threat in healthcare-associated infections, especially causing deadly lung infections in immunocompromised or cystic fibrosis patients. As other opportunistic pathogens, *B. cenocepacia* employs lectins, i.e., carbohydrate-binding proteins, as virulence factors responsible for recognition of and adhesion to glycoconjugates on the host cell surface, and subsequent infection process (Poole et al., 2018). Disrupting the binding of lectins to host oligosaccharides is increasingly seen as a suitable approach to hinder microbial adhesion and prevent the bacterial infection at its onset. This strategy, known as anti-adhesion therapy (AAT), is expected to complement conventional antibiotic treatments and to reduce the appearance of resistant strains (Gerling-Driessen et al., 2023). In this context, we recently envisioned *B. cenocepacia* BC2L-C lectin as a candidate target for AAT against this pathogen. BC2L-C has been proposed as a major player for bacterial adhesion and biofilm formation because it can mediate crosslinking between *B. cenocepacia* surface and human epithelial cells by simultaneously binding bacterial heptosides and human fucosides (Šulák et al., 2011). Indeed, this lectin presents a double carbohydrate specificity through a hexameric architecture, displaying a mannose-specific C-terminal dimeric domain and a fucose-specific N-terminal trimeric domain, which makes it a superlectin (Šulák et al., 2010, 2011). In our initial effort, we identified BC2L-C N-terminal domain (BC2L-C-Nt) as a relevant target to design glycomimetic antagonists for AAT to prevent lectin-mediated bacterial adhesion to the host epithelium. The trimeric N-terminal domain shows millimolar affinity for α-methyl-L-fucoside and high micromolar affinity for fucosylated histo-blood oligosaccharides (Šulák et al., 2010, 2011; Bermeo et al., 2020). We also found that BC2L-C-Nt binds L-galactose with an affinity which is similar to that of α-methyl fucoside (Bermeo et al., 2020).

**FIGURE 1:**
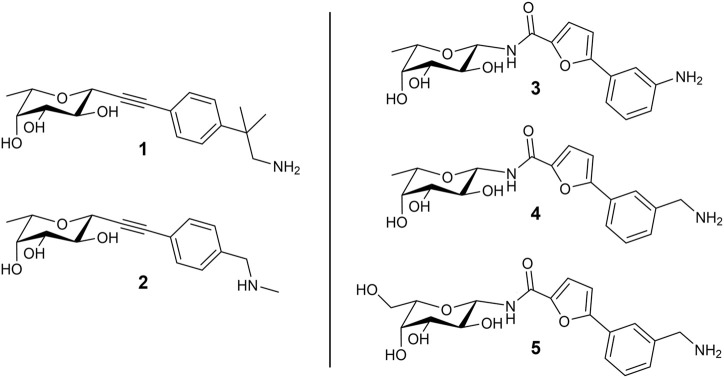
Structure of the bifunctional glycomimetics 1–5. Ligands 1-4 contain L-fucose (6-deoxy-L-galactose) as a monosaccharide anchor; ligand 5 contains L-galactose. Compounds 1 and 2 are characterized by an alkyne linker, while compounds 3-5 contain an amide linker.

In a previous study, we used the BC2L-C-Nt complex with α-methylselenyl-fucoside (PDB 2WQ4) to identify by virtual screening a set of fragments able to occupy a secondary site at the interface of two monomers, in the vicinity of the fucose binding site. The fragments that were validated by biophysical techniques are mostly constituted by an aromatic moiety, predicted to interact with residue Tyr58 in the vicinal site through T-shaped π-interactions. Additionally, some fragments are endowed with a terminal amino group that is predicted to engage residue Asp70 at the bottom of the secondary site through ionic or polar interactions (Lal et al., 2021). Using these fragments, we rationally designed and screened *in silico* by molecular docking a set of bifunctional β-C- and β-N-fucosides, generated by connecting the fragments to the anomeric carbon of L-fucose through suitable linkers. Bifunctional molecules able to simultaneously occupy both the sugar binding site and its vicinal region were identified, and some of them were synthesized and tested against their target in two successive campaigns (Bermeo et al., 2022; Mazzotta et al., 2023). The first BC2L-C-Nt synthetic ligands showed up to a 10-fold affinity gain over the parent monosaccharide, as determined by isothermal titration calorimetry (ITC), and resulted in the first three crystal structures of antagonist/BC2L-C-Nt complexes. These data validated a few chemotypes as glycomimetic bifunctional ligands for BC2L-C-Nt, including β-fucosyl alkynes, β-fucosyl and β-L-galactosyl amides. The structures of five representative ligands comprising some among the most active non-natural ligands described so far against BC2L-C-Nt, are collected in Figure 1. In these molecules, the monosaccharide ring acts as an anchor, able to drive the ligand to the lectin binding site, and the fragment-derived element provides increased affinity (and possibly selectivity) for the target (Bernardi and Sattin, 2020).

The computational work based on flexible ligand/rigid protein docking calculations proved to be an appropriate tool for fragment selection and rational design of glycomimetic structures, providing insights in the binding poses of the ligands. However, some peculiarities of protein-carbohydrate interactions may be inappropriately accounted for by docking programs and scoring functions (Pérez and Tvaroška, 2014). In particular, the role played by desolvation of both the carbohydrate ligands and the protein carbohydrate binding site is not explicitely considered in docking protocols, but it is certainly of paramount importance for these highly solvated moieties. It has been noted that docking of carbohydrates and glycomimetics into proteins may tend to maximize interactions between ligand and receptor, and the resulting poses may show the carbohydrate ligand in close contact with protein residues. However, X-ray crystal structures frequently display different features, with some moieties adopting different orientations or extending towards the solvent, or interacting through water-mediated contacts. These structures might be more accurately computed if solvation (e.g., water molecules competing for hydrogen bonds) is taken into account. Another significant limitation in docking is that it is typically performed while keeping the protein rigid. Molecular Dynamics simulations in explicit water have been extensively and successfully applied to validate and refine binding modes obtained from automated molecular docking (Pérez and Tvaroška, 2014). Energy analyses with the Poisson–Boltzmann and generalized Born solvent models (MM-PB/GBSA) have also been used to account for solvation effects (Sood et al., 2018). In this work the interplay of several computational methods is investigated and the results compared with experimental data, in an effort to define a practical protocol for the structure-based design of glycomimetic ligands. Therefore, with the aim of unravelling the molecular basis of ligand binding and looking into the dynamic traits of glycomimetic/lectin complexes, we performed all-atom Molecular Dynamics (MD) simulations in explicit solvent of the ligands shown in Figure 1 in complex with the trimeric structure of BC2L-C-Nt. Insights into the flexibility of the binding interface and the interactions of both the sugar and the non-sugar part of the ligands with lectin residues were gained, together with information about the role of the solvent. Molecular Mechanics Generalized Born Surface Area (MM-GBSA) analyses were performed to re-score and select docking poses, and to evaluate binding free energies of the complexes on the calculated trajectories. Here the computational results are reported and compared with experimental data from several biophysical techniques, including ITC and X-ray crystallography. This study provided new relevant information for the structure-based design of improved BC2L-C-Nt ligands as novel antimicrobials with antiadhesive properties.

## 2 Materials and methods

### 2.1 Computational studies

All the calculations were performed using the Schrödinger Suite through Maestro graphical interface (Schrödinger Release 2018–1 and 2021–1).

### 2.2 Ligand preparation

The glycomimetic bifunctional ligands were prepared for docking using the LigPrep tool to create energy minimized 3D structures. The protonation states were generated at pH 7 ± 2. Neutral aniline for ligand 3 and protonated amino groups for the other ligands were suggested as the most favorable protonation states and then employed in computational studies.

### 2.3 Protein preparation

Atomic coordinates from the crystal structure of BC2L-C-Nt in complex with MeSe-α-L-Fuc (PDB 2WQ4) were taken from the Protein Data Bank (Šulák et al., 2010). The asymmetric unit comprises three peptide chains (A, B, C) and three carbohydrate ligands (MeSe-α-L-Fuc), around a 3-fold pseudo axis of symmetry. The crystal structure reveals three fucose binding sites located at the interface between neighboring monomers, and an identical binding mode for the sugar in the three binding sites. In each of them, key residues from one chain (Tyr48, Ser82, Thr83, Arg85) and from the neighboring chain (Tyr58, Thr74, Tyr75, Arg111) play an important role an ligand binding. In addition, two water molecules, HOH2195 (w1) and HOH2194 (w2), bridge the sugar and the protein. As both water molecules are conserved in the available crystal structures of BC2L-C-Nt in complex with fucosylated oligosaccharides and glycomimetic ligands, they were retained in the trimeric protein structure set at this step, in accordance with our previous docking calculations. The system was prepared using the Protein Preparation Wizard of the Maestro graphical user interface. The hydrogen atoms were added and pKa was calculated for protein residues using the PROPKA method (Olsson et al., 2011) at pH 7.4. The HIE protonation state was also assigned to histidine (His116) residue. Then, the protein-ligand complex was subjected to restrained minimization with convergence of heavy atoms to an RMSD of 0.3 Å using the OPLS3 force field (Harder et al., 2016). The final structure was used to generate the grid for docking calculations.

### 2.4 Docking calculations

Docking calculations were performed using Glide (Grid-based Ligand Docking with Energetics) (Friesner et al., 2004) version 7.8.

The docking grid was prepared removing the MeSe-α-L-Fuc residue located between chains A and C, while retaining the crystallographic ligand in the other two binding sites between chains A and B, and between chains B and C. The two water molecules (w1 and w2) mentioned above were also retained. The centroid of the fucoside located in the active site between chain A and chain C (considered as the ligand in the grid generation protocol), was used to define a cubic grid inner box with dimensions 10 × 10 × 10 Å and a cubic grid outer box with dimensions 20.2 Å. Docking calculations were carried out applying the flexible docking approach and employing both the extra precision (XP) and the standard precision (SP) scoring function with the OPLS3 force field. No Epik state penalties were added to the final docking scores.

The selenium atom of the fucoside in the crystal structure was replaced by oxygen and the α-methylfucoside obtained was redocked at the sugar binding site. The program reproduced the co-crystallized binding mode of the fucoside with an RMSD value of 0.48 Å, thus validating the docking protocol. The bifunctional glycomimetics were analyzed employing the same docking protocol and saving at most 10 poses using the SP and XP scoring function.

### 2.5 MD simulations

MD simulations were carried out using Desmond (Desmond, Schrodinger release 2021–1, Bowers et al., 2006) version 6.5 in NPT conditions setting T = 300K and *p* = 1 atm using the Langevin thermostat and barostat (Grest et al., 1986) with relaxation time set to 1.0 ps and 2.0 ps, respectively. Docking poses of ligands in the trimeric BC2L-C-Nt structure were considered as starting structures; after removing the crystallographic water molecules w1 and w2, each docking pose was solvated with a truncated octahedral TIP3P (transferable intermolecular potential 3-point) (Mark and Nilsson, 2001) water box of 12 Å. The system was neutralized by adding the proper number of Cl- ions, and NaCl salt (0.15 M) was also added to recreate a physiological environment. The systems were equilibrated by applying the ‘desmond_npt_relax.msj’ protocol available in Desmond with the default parameters and the OPLS4 force field (Lu et al., 2021). The integration time step was set to 2 fs, and a 9 Å cutoff radius was chosen for the short-range Coulomb interactions within the u-series decomposition of the Coulomb potential (Predescu et al., 2020). For each ligand two simulations of 500 ns were carried out saving 5000 structures from each run for the analysis.

The ‘Simulation interactions diagrams’ and ‘Simulation Event analysis’ tools of Desmond were employed for the analysis of the trajectories and for the evaluation of the stability of the system. Protein dynamics was assessed by RMSD (backbone atoms) and RMSF (Cα atoms only) analysis. The trj_occupancy.py script was executed on trajectories to calculate the occupancy histogram of water atoms in 3D space around the glycomimetic ligands. The trajectory was aligned to the first frame on the ligand atoms and the occupancy was calculated on water atoms (res.ptype T3P) for a cubic subspace (grid spacing 1 Å, grid length 20 Å) centered at the ligand center-of-mass. The result is written into a file in the cns map format for visualization on Maestro.

### 2.6 MM-GBSA calculations

The MM-GBSA (Molecular Mechanics Generalized Born Surface Area) binding energy was calculated employing Prime software (Jacobson et al., 2002, 2004) (Prime MM-GBSA version 3.0) using the VSGB2.0 (Li et al., 2011) implicit water model and the OPLS4 force field. Docking poses were used for MM-GBSA calculations and all protein atoms were retained during calculations. The thermal_mmgbsa.py script was executed to calculate the MM-GBSA binding energy on frames extracted from the trajectories of MD simulations. 1000 frames were selected from each trajectory (every 5th frame from total 5000 frames), and the MM-GBSA calculations were performed on them after deleting waters and separating the ligand from the receptor.

## 3 Results and discussion

### 3.1 Docking of bifunctional glycomimetics

The structures of the BC2L-C-Nt glycomimetic ligands considered in this work (1–5) are shown in Figure 1. They belong to the recently reported panel of first-generation ligands for this lectin domain, which comprise bifunctional β-C- and β-N-fucosides (Bermeo et al., 2022; Mazzotta et al., 2023) containing a fucose or fucose-like core connected in the anomeric position to fragments aimed at engaging a secondary site of the lectin, in the vicinity of the fucose binding site. In particular, compounds 1 and 2 are β-L-fucosyl alkynes characterized by a triple bond as a linker between the sugar moiety and the fragment portion, while the other compounds employ an amide bond as a linker, affording β-L-fucosyl and β-L-galactosyl amides. This small set of ligands includes some of the glycomimetics with the highest affinity for BC2L-C-Nt described so far, as measured by ITC. The K_D_ values determined by ITC experiments with BC2L-C-Nt are collected in Table 1, along with the values measured for the parent monosaccharides. The hit compound 4 exhibits good water solubility and an affinity for BC2L-C-Nt of 159 μM, which represents a one order of magnitude gain over α-methyl fucoside. The affinity of the closely related 3 could not be determined by ITC, because of the low water solubility of this ligand, but an X-ray structure of its BC2L-C-Nt complex was obtained at 1.32 Å resolution (PDB code 7OLW) that fully supported the design criteria and the docking pose. The binding affinities of the L-galactosyl derivative 5 (K_D_ 390 μM) and of the L-fucosyl alkyne 1 (K_D_ 280 μM) are only slightly lower than the hit. These data confirm that BC2L-C-Nt can accommodate a hydroxyl group on C6 of the monosaccharide (e.g., 5 vs. 4) on one side, and different aglycone moieties (e.g., 1 vs. 4) on the other, although small changes in the aglycone portion can lead to a significant decrease in affinity (e.g., 2 K_D_ 1.24 mM vs. 1 K_D_ 280 μM). The X-ray structures of the complexes of 1 and 4 with BC2L-C-Nt were also solved at good resolution (1.79 Å and 1.55 Å, PDB code 7OLU and 8BRO, respectively), affording further structural information on the binding mode of these bifunctional ligands.

**TABLE 1:** Affinity of carbohydrate ligands (bifunctional glycomimetics 1-5 and parent monosaccharides) to BC2L-C-Nt by ITC.

Compound	ITC K_D_ (mM)	References
1	0.28 ± 0.01	Bermeo et al. (2022)
2	1.24 ± 0.07	Bermeo et al. (2022)
3	N.D.	Bermeo et al. (2022)
4	0.159 ± 0.007	Mazzotta et al. (2023)
5	0.390 ± 0.015	Mazzotta et al. (2023)
α-methyl-L-Fuc	2.700 ± 0.007	Šulák et al. (2010)
L-Gal	2.00	Bermeo et al. (2020)

**FIGURE 2:**
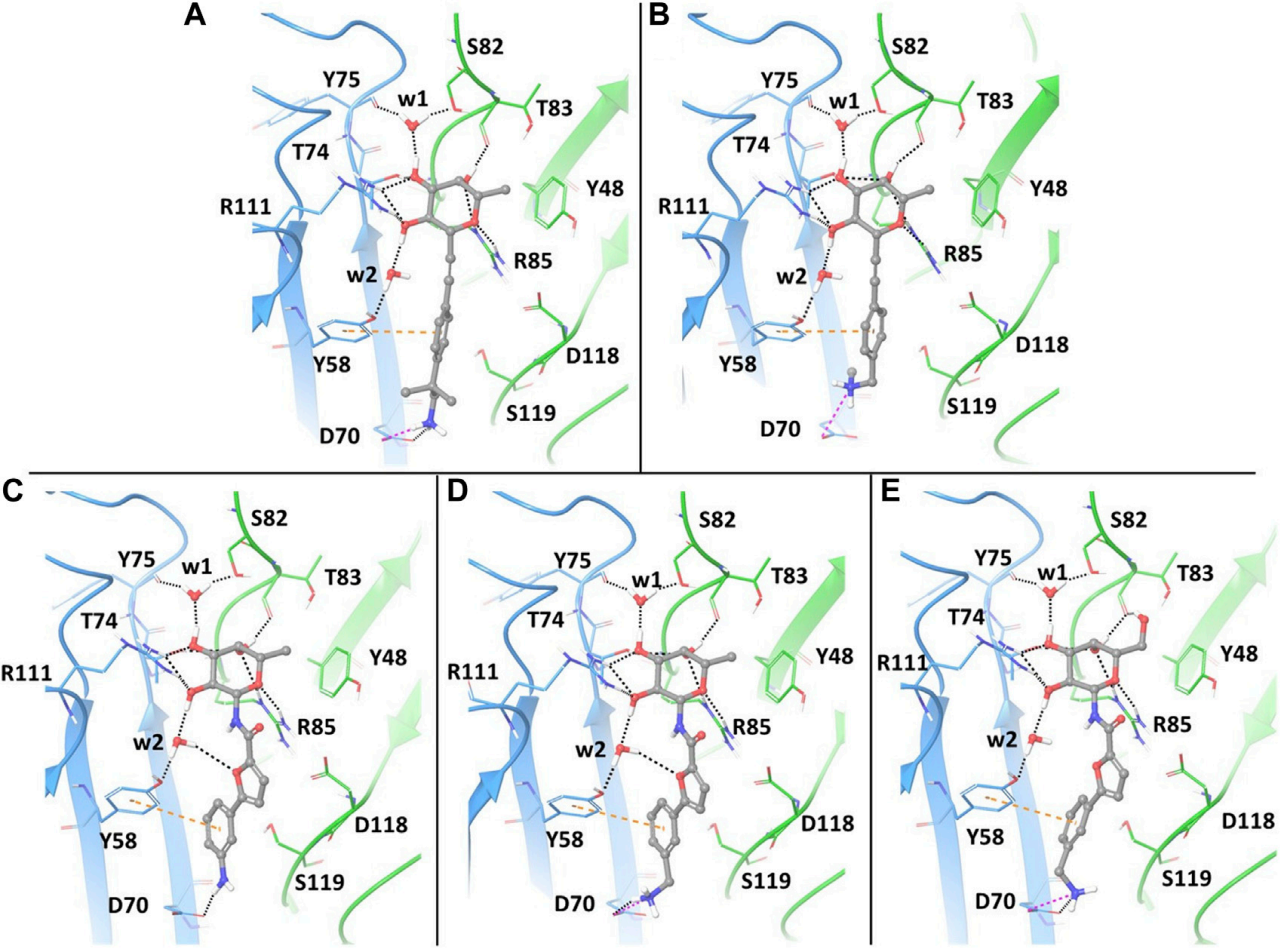
Docking ligands 1-5 in PDB 2WQ4: best poses, as assessed by the XP scoring function of Glide. The chain C of the protein is shown in light blue and chain A in green. The ligands are colored according to atom type. The retained water molecules w1 and w2 are shown as red spheres. The interactions are represented with dotted lines: black for H-bonds, pink for salt bridges and orange for π- π stacking interactions. **(A)** complex with ligand 1; **(B)** complex with ligand 2; **(C)** complex with ligand 3; **(D)** complex with ligand 4; **(E)** complex with ligand 5.

With the aim of gaining insights on the dynamics of the complexes, we performed extensive MD simulations for all five complexes, using the full protein trimer. First, we identified suitable starting structures for all atom simulations among the docking poses. Docking of the bifunctional glycomimetics was performed in the trimeric structure of the BC2L-C-Nt, centering the grid in the active site between chain A and chain C, as described in the Material and Methods section. A molecule of α-Me fucoside was retained in the other two binding sites of the trimer. Two crystallographic conserved water molecules, w1 and w2 (see Figure 2), that mediate interactions between the protein and the fucose ring were also retained in the docking model. Docking poses from both the SP and the XP protocols of Glide were analyzed in detail, evaluating the ability of the fucose (or fucose-like) core of each ligand to adopt the binding mode observed in available crystal structures, as well as the ability of the aglycone portion to establish stabilizing interactions with lectin residues. The analysis revealed that docking calculations using the XP scoring function are more effective than runs employing the SP protocol in reproducing the binding mode of the fucose core observed in the crystal structures, especially for amide derivatives 3-5.

The best poses of the five glycomimetics according to the XP scoring function are displayed in Figure 2. The sugar portion of all ligands directly interacts with residues Thr83 and Arg85 from chain A, and Thr74 and Arg111 from chain C, as observed in the available crystal structures. Interactions between Fuc-OH 3 and Tyr75/Ser82 and between Fuc-OH 2 and the side chain of Tyr58 from chain C are mediated by water w1 and w2, respectively (Figures 2A–E). The L-galactose moiety in compound 5 forms additional interactions through the OH group in position 6, e.g., by H-bonding with the backbone carbonyl moiety of Thr83 (Figure 2E).

Regarding the fragment portion of the ligands, T-shaped π- π stacking interactions are observed involving the aromatic moiety of the glycomimetics and residue Tyr58 from chain C. Additionally, the terminal amino group of compounds 1, 2, 4 and 5 (Figures 2A, B, D, E, respectively) is predicted to form a salt bridge with the Asp70 side chain at the bottom of the vicinal site, while only H-bond interactions between the same residue and the aniline moiety are possible for compound 3 (Figure 2C). The calculated docking poses mainly differ for the conformation of the short carbon chain connecting the amino group to the aromatic moiety in the fragment portion, or for the orientation of the OH group in position 6 of galactose.

**FIGURE 3:**
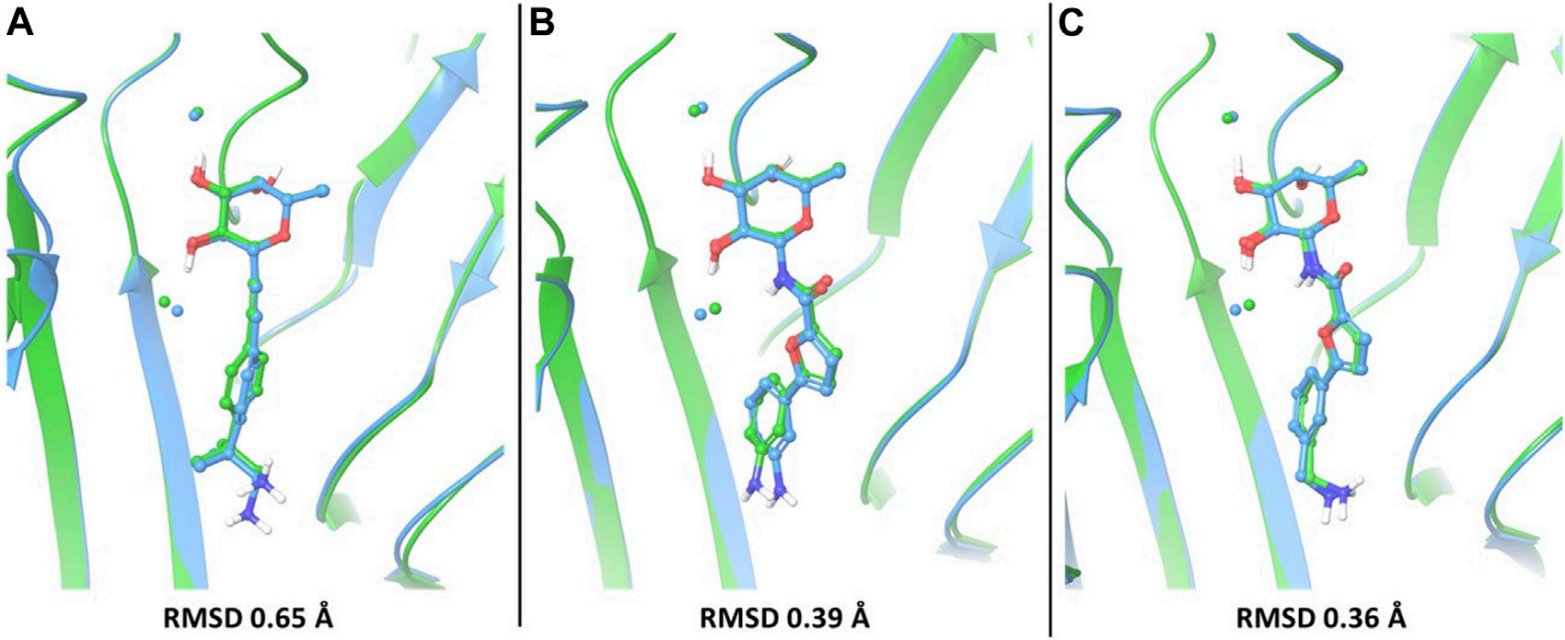
The best-fit docking poses (light blue) are superimposed to X-ray structures (green) for compounds 1 **(A)**, PDB 7OLU), 3 **(B)**, PDB 7OLW) and 4 **(C)**, PDB 8BRO). For each of these compounds, the third-ranked docking pose has the best fit with the experimental binding mode and is shown here. Proteins were aligned by protein binding site alignment, then RMSD was calculated on the heavy atoms of ligand in-place. The two conserved water molecules (w1 and w2) are depicted as spheres: green for the X-ray structures and light blue for the docking pose.

In particular, the docking poses of compounds 1, 3 and 4 show interaction patterns similar to those observed in the corresponding crystal structures with BC2L-C-Nt (PDB code 7OLU, 7OLW and 8BRO) (Bermeo et al., 2022; Mazzotta et al., 2023). Figure 3 shows the overlap between the X-ray structure (green) and the best-fit docking pose (light blue) for these compounds. Curiously, in all cases this pose is ranked number 3 by the XP scoring function. For amide 3, there is very little difference between this pose (Figure 3B) and the one scored lowest by the docking algorithm and shown in Figure 2C. Indeed, the RMSD values relative to the X-ray structure are 0.48 Å for pose 1 and 0.39 Å for the best fit pose # 3. For amide 4, the difference is similarly small (RMSD value of 0.51 Å for pose 1 and 0.36 Å for pose 3). As it can be appreciated by comparing Figure 2D with Figure 3C, the main difference between the two poses is the location of the terminal amino group, which in pose 1 (Figure 2D) has a direct interaction with Asp 70. For the β-fucosyl alkyne 1 the best fit with the experimental binding mode is also provided by the third-ranked docking pose (RMSD value of 0.65 Å), but this differs more significantly from the best XP pose (RMSD 1.40 Å). Inspection of Figure 2A (lowest XP pose) and Figure 3A (X-ray structure and pose #3) shows that the two differ by an approximately 90° rotation around the benzylic bond, which allows one of the methyl groups on the benzylic carbon of 1 to be buried into the crevice formed at the interface of protein monomers, as experimentally observed by X-ray crystallography. Apparently, the complex stabilization resulting from these additional van der Waals interactions is not fully appreciated by the XP scoring function. It is also worth noting that, instead of the salt bridge predicted by docking, a water-mediated contact between Asp70 and the amino group is observed in the crystal structures of ligands 1 and 4 with BC2L-C-Nt.

### 3.2 Re-scoring and selection of poses by MM-GBSA calculations

The docking poses were re-scored employing the Prime MM-GBSA method (Hou et al., 2011). It consists in assessing the free energies of ligand-protein complexes in implicit solvent as the difference between the energy of the bound complex and the energy of the unbound protein and ligand (Rastelli et al., 2010). In this work, MM-GBSA calculations were performed to analyze the various binding poses generated by docking and select the most stable ones as starting structures for the MD simulations. The MM-GBSA method is also widely used for re-scoring different possible ligands identified by virtual screening of compound libraries, even in consensus strategies (Lyne et al., 2006; Sirin et al., 2014; Zhan, 2017). Indeed, scoring functions represent the most critical factor in determining the overall reliability of docking approaches.

**FIGURE 4:**
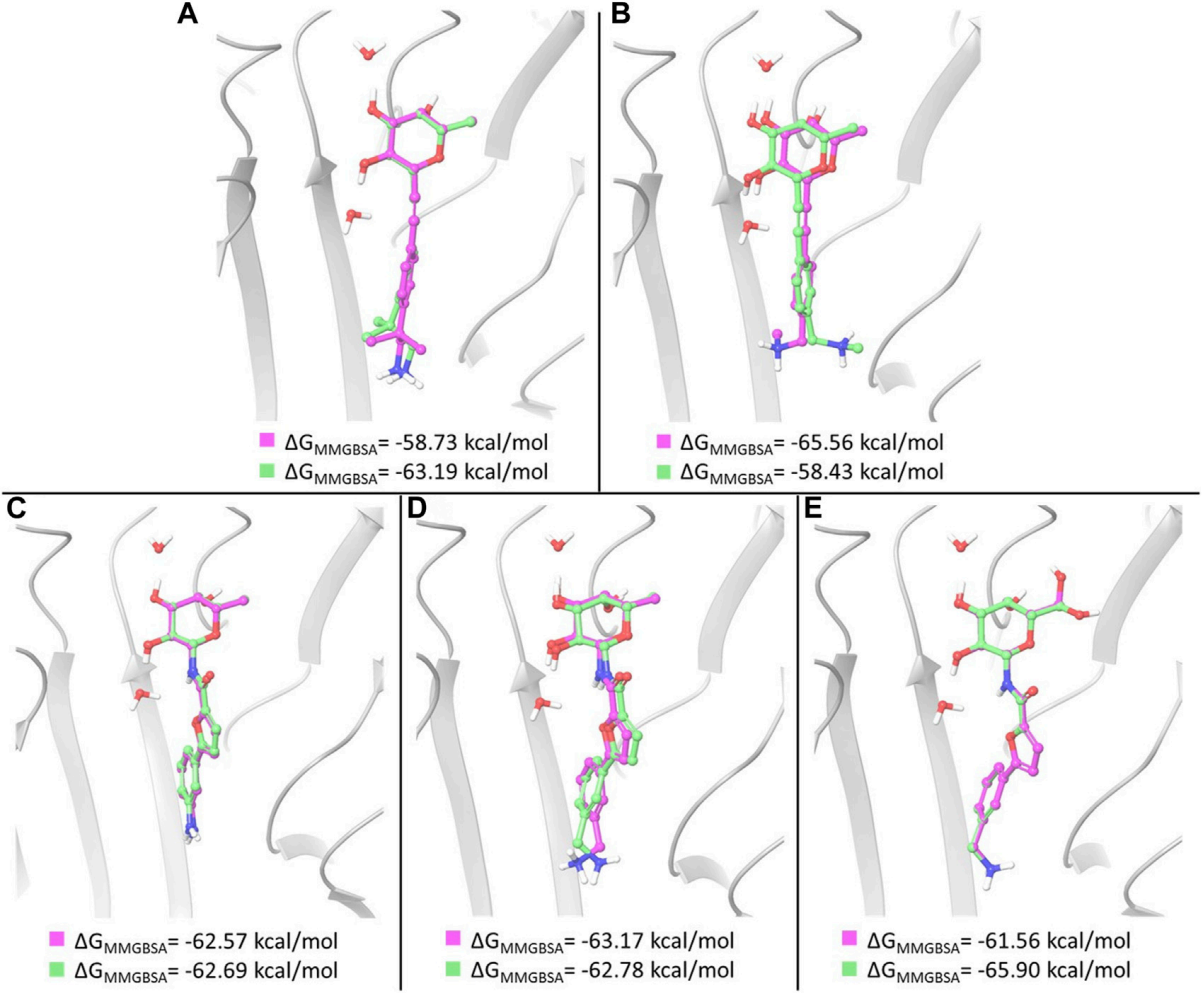
Overlay of the two poses selected as starting structures for MD simulations with ligand 1 **(A)**, 2 **(B)**, 3 **(C)**, 4 **(D)** and 5 **(E)**. In pink, the pose with the lowest XP score, in green the other selected pose. ΔGMM-GBSA values calculated for the poses are reported.

In the case of amides 3 and 4, whose X-ray structures in complex with BC2L-C-Nt are available, MM-GBSA free energy calculations basically confirm the ranking of docking poses provided by the XP scoring function. In particular, the three top ranked poses of ligand 3, which are very similar to one another and to the X-ray structure, were found to have comparable MM-GBSA energy values as well. Thus, the first and third docking poses discussed above show the lowest MM-GBSA energy values and were selected for MD simulations (Figure 4C). In the case of ligand 4, the ranking of the three top poses is also confirmed by MM-GBSA calculations. The first and third pose discussed above, that differ in the placement of the benzylamine moiety, were selected as starting structures for MD simulations (Figure 4D).

For ligand 1, a different ranking order of the poses is provided by MM-GBSA free energy calculations. In particular, it is worth noting that the MM-GBSA method recognizes as the lowest-energy pose the experimental (X-ray) binding mode observed in the pose ranked #3 by the XP scoring function. As observed above, an important feature of this binding mode is represented by the hydrophobic complementarity established by one methyl group of the fragment with a suitable hydrophobic portion on the protein surface. An MM-GBSA energy difference of 4.5 kcal/mol is calculated after re-scoring between the third and the first docking pose. These poses, differing in the orientation of methyl and amino groups in the fragment portion (Figure 4A), have been selected as starting structures for MD simulations.

Two representative docking poses have been identified also for ligands 2 and 5, based on their MM-GBSA energies. The top ranked poses of ligand 2 differ in the orientation of the terminal amino group (Figure 4B), while the poses selected for ligand 5 show a different arrangement of the 6-OH group of L-galactose. Even in the latter case a different ranking of docking poses is provided by MM-GBSA free energy calculations where the third docking pose displays the lowest MM-GBSA energy (Figure 4E).

### 3.3 MD simulations of glycomimetic/BC2L-C-Nt complexes

The poses selected as described above were used as starting structures in MD simulations carried out employing the Desmond package (Desmond, Schrodinger release 2021–1). MD simulations are a powerful and useful technique for a comprehensive analysis of the biomolecular dynamics. In particular, they can provide detailed information on the flexibility of a protein and on the interaction dynamics of protein–ligand complexes, by monitoring atomic position deviations and interactions over time. Moreover, MD simulations allow to investigate the stability of the binding mode observed for a ligand in the docking pose. Compared to X-ray structures, they can also provide a description of the ligand-receptor interaction that occurs in a dynamic situation, as in the case of a binding event in solution, thus helping to rationalize experimental binding data. The dynamics of all five complexes were run for 2 × 500 ns, starting from two different poses. The crystallographically conserved water molecules w1 and w2 were removed, before solvating the complexes with TIP3P water and equilibrating, as detailed in the Material and Methods section.

**FIGURE 5:**
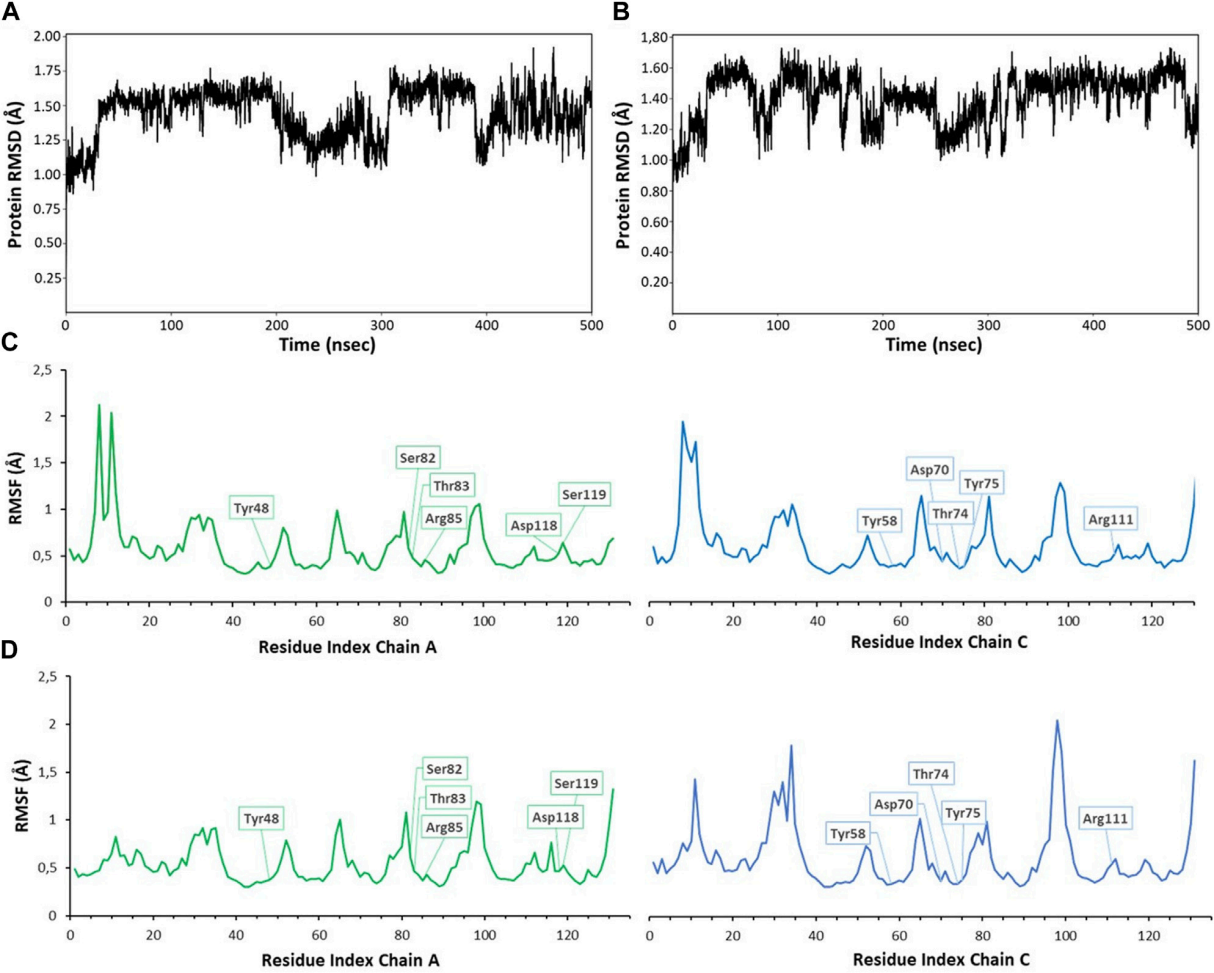
RMSD (root mean square deviation) and RMSF (root mean square fluctuation) values calculated for 500 ns MD simulations of compound 1 **(A,C)** and 4 **(B,D)**. RMSD values were calculated on backbone protein atoms after alignment of trajectory frames to the first frame **(A)**, plot for ligand 1; **(B)**, plot for ligand 4). RMSF per-residue was calculated on Cα atoms, always considering the first frame as reference structure [**(A)** chain in green, **(C)** chain in blue]. The residues interacting with ligands are highlighted in boxes **(C)**, plot for ligand 1; **(D)**, plot for ligand 4). Similar plots were obtained for other compounds and simulations. RMSD plots were obtained using the ‘Simulation interactions diagrams’ tool of Desmond.

Monitoring the RMSD (root mean square deviation of the atomic positions) evolution of the protein throughout the simulation provides information on global conformational changes, while analysing the root mean square fluctuation (RMSF) for individual residues is useful to characterize local changes along the protein chain. Small variations of protein RMSD values, ranging between 1 and 2 Å, are observed in all simulations (RMSD values from MD simulations with ligands 1 and 4 are shown in Figures 5A, B), suggesting the lack of large conformational changes in the protein trimer. Since the simulations were performed on the trimeric structure of BC2L-C-Nt in complex with the bifunctional glycomimetic at the binding site between monomer A and C and with α-methyl fucoside in the other two binding interfaces, the analysis of the RMSF values focused on the residues belonging to chain A and C. As an example of the observed trends, the RMSF values for residues of monomer A and C from MD simulations with ligands 1 and 4 are shown in Figures 5C, D. The plots show that residues at the binding interface (e.g., Tyr48, Ser82, Thr83, Arg85, Asp118 and Ser119 from chain A and Tyr58, Asp70, Thr74, Tyr75 and Arg111 from chain C) display the lowest fluctuations, proving the stability of the monomer interface involved in ligand binding during the simulation. Slightly larger fluctuations are observed at N-terminal residues and at the level of the surface loops, particularly for loop Val28-Asp35, Gly51-Pro55, Val96-Val100, whose conformational flexibility was already apparent in the comparison of different crystal structures (Lal et al., 2021).

**FIGURE 6:**
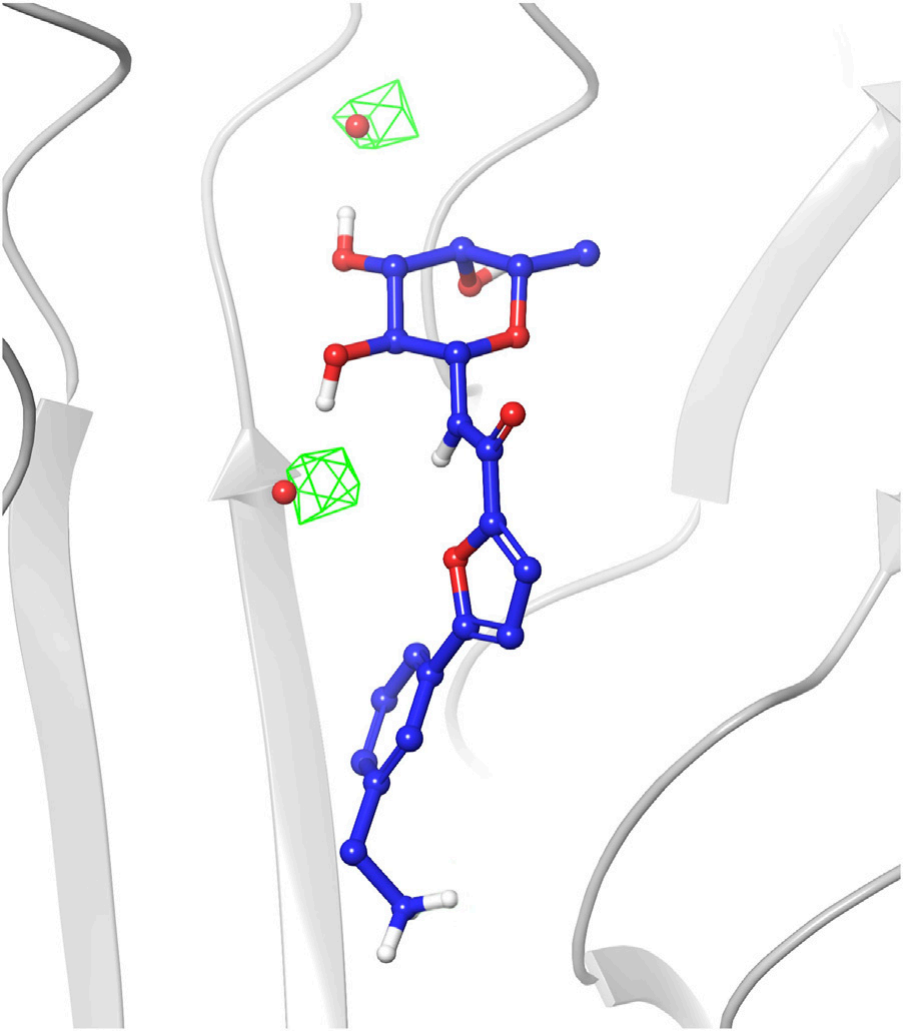
Occupancy map of water atoms in 3D space around ligand 4 calculated on the trajectory of the MD simulation from the third-ranked docking pose. The trajectory was aligned to the first frame on the ligand atoms, then the occupancy was calculated on water atoms using the trj_occupancy.py script. The result is shown as green isosurfaces at sigma values of 6.86 superimposed on the x-ray structure (crystallographic water w1 and w2 shown as red spheres, PDB 8BRO).

The analysis of the structures sampled for ligands 1-4 confirmed that the fucose moiety stably retains the arrangement observed in the crystal structures. Analysis of the RMSD values calculated on ligand heavy atoms of the fucose moiety using the first frame as reference structure reveals that the sugar starting conformation is preserved during the entire simulations and only small oscillations are noticed in the plots (average RMSD values in the range 0.11–0.17 Å, Supplementary Figure S1). The sugar maintains all key interactions with the protein throughout the simulations, such as direct hydrogen bonds with Arg111 and Thr74 from chain C, and with Thr83 and Arg85 from chain A. Water-mediated interactions with Tyr75, Tyr58 and Ser82 were also conserved (see Supplementary Figure S2 in Supplementary Materials for a detailed analysis of the interactions). The latter observation highlights that during the simulations TIP3P water molecules indeed occupy similar areas as the two water molecules conserved in crystal complexes and retained in the docking studies as w1 and w2, establishing the same interaction pattern. This is also visible in the water occupancy maps (Figure 6) calculated from MD trajectories as described in the Material and Methods section. The regions with the highest water occupancy are located around the crystallographic w1 and w2 water molecules, as shown in Figure 6 for one of the simulations with ligand 4. In particular, one of the two highest-occupancy isosurfaces is perfectly centered on w1, that is deeply buried in the binding site, while the second one is slightly shifted relative to the position of w2 in the X-ray complex with ligand 4 (PDB code 8BRO). Indeed, this second water molecule is more exposed than w1 to the bulk solvent and its less-defined position is already apparent when comparing different crystal structures of BC2L-C-Nt complexes.

According to the simulations’ results, the L-galactose core of compound 5 can establish the same interactions already observed for fucose, but in addition the hydroxyl group in position 6 can be involved in direct and water-mediated hydrogen bonds with adjacent residues, such as Arg85 and Thr83. That these additional interactions do not result in an increased affinity of 5, relative to 4, is clear from the ITC data of Table 1. However, they allow L-galactose to be accommodated in the fucose binding site, despite the loss of hydrophobic interactions in the methyl-binding groove, and expand the chemical space exploitable for the design of BC2L-C antagonists. The 6 OH moiety of galactose has a higher mobility compared to the other OH groups of the sugar, thus explaining the larger RMSD value of galactose heavy atoms (average RMSD value 0.28 Å, Supplementary Figure S1) relative to the values calculated for fucose.

In general, the aglycone portion of the ligands showed larger flexibility compared to the sugar part, as it contains a flexible side chain that can rotate and adopt different conformations. Compound 3 is an exception, because it comprises an aniline moiety, thus resulting more fixed and rigid within the binding site. This trend is well illustrated in the plots of RMSD values calculated for the fragment part of ligands during each simulation (Supplementary Figure S3). β-fucosyl alkynes 1 and 2 are the most flexible of the structures examined, and dynamically sample a larger range of poses for their aglycone portion (average RMSD values 0.89 Å and 0.82 Å, respectively, Supplementary Figure S3). In particular, the benzylic side chain of ligand 1, while maintaining the amino group involved in the H-bond interactions described below, rotates freely sampling all possible conformations around the benzylic bond (see movie in Supplementary Material). This allows one of the geminal methyl groups to occupy the underlying hydrophobic cleft during more than half of the simulation.

**FIGURE 7:**
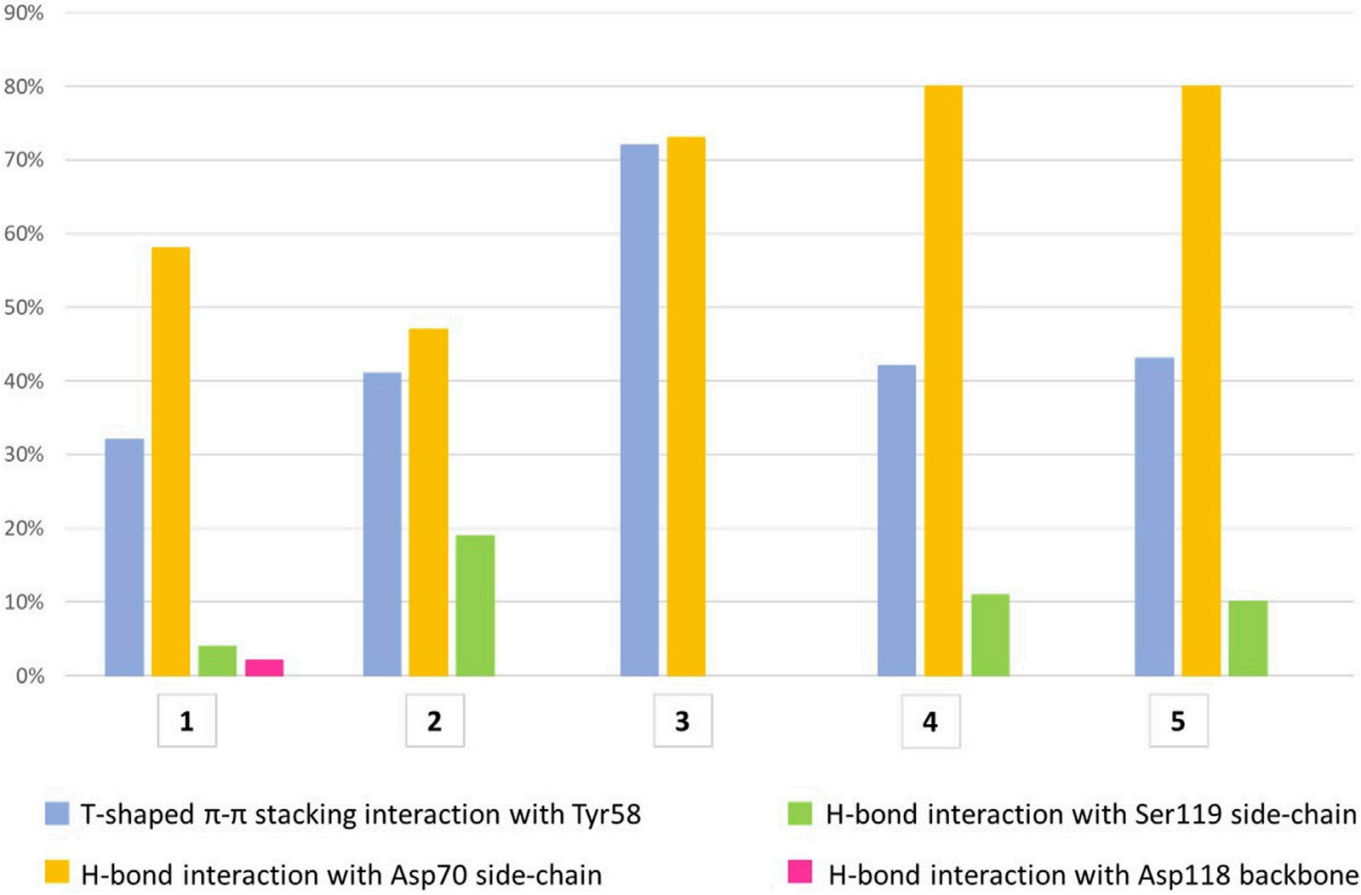
The histograms represent the interaction of the aglycone portion in the binding crevice. T-shaped π-π stacking interactions with Tyr58 (light blue) and H-bonds with Asp70 (yellow), Ser119 (green) and Asp118 (pink). The values plotted are averages of percentages assessed over two 500 ns simulation runs of each ligand. The T-shaped π-π stacking interaction is established when the distance between the centroids of the rings is ≤5.5 Å and the angle between the ring planes is ≥30°. The geometric criteria for protein-ligand H-bonds are the following: a distance ≤2.5 Å between donor H atom and acceptor atoms (D—H···A); a donor angle ≥120° between the donor-hydrogen-acceptor atoms (D—H···A); an acceptor angle ≥90°between the hydrogen-acceptor-bonded_atom atoms (H···A—X).

All compounds were able to establish T-shaped π-π stacking interactions during simulations, involving the aromatic ring of the ligands and the side chain of Tyr58 from chain C (Figure 7, light blue bars). According to the simulation results, this interaction is observed in the 30%–45% of the saved structures, except for compound 3, for which it is detected in about 72% of the sampled structures. This agrees with the reduced flexibility of ligand 3, which retains a binding mode very similar to that of the starting structure throughout the simulation.

The hydrogen bond interactions formed by the terminal amino group were also monitored during the simulations. For all ligands, the main partner of interaction is the carboxyl group in the side chain of Asp70 from chain C. As shown in Figure 7 (yellow bars), compounds 3-5 establish direct H-bond interactions with Asp70 more easily than ligands 1 and 2, likely due to the increased length of the aglycone portion. To a lesser extent, all ligands except 3 form direct H-bond interactions with Ser119 from chain A, whose side chain hydroxyl moiety can interact with the ligand amino group (Figure 7, green bars). In particular, ligand 2 displays the highest percentage of H-bond interactions with Ser119 among all the molecules investigated, suggesting an increased mobility within the binding site, which allows its terminal amino group to fluctuate between Asp70 and Ser119. This could lead to an unstable interaction with the target protein explaining the lower affinity assessed by ITC assay for this molecule relative to the closely related 1, whose amino group is stably connected to Asp70 (yellow bar, 58%) and only rarely interacts with Ser119 (4%, green bar). Additionally, direct H-bond interactions with the main chain oxygen of Asp118 from chain A are displayed by ligand 1, but only in 2% of the sampled structures (Figure 7, pink bar).

**FIGURE 8:**
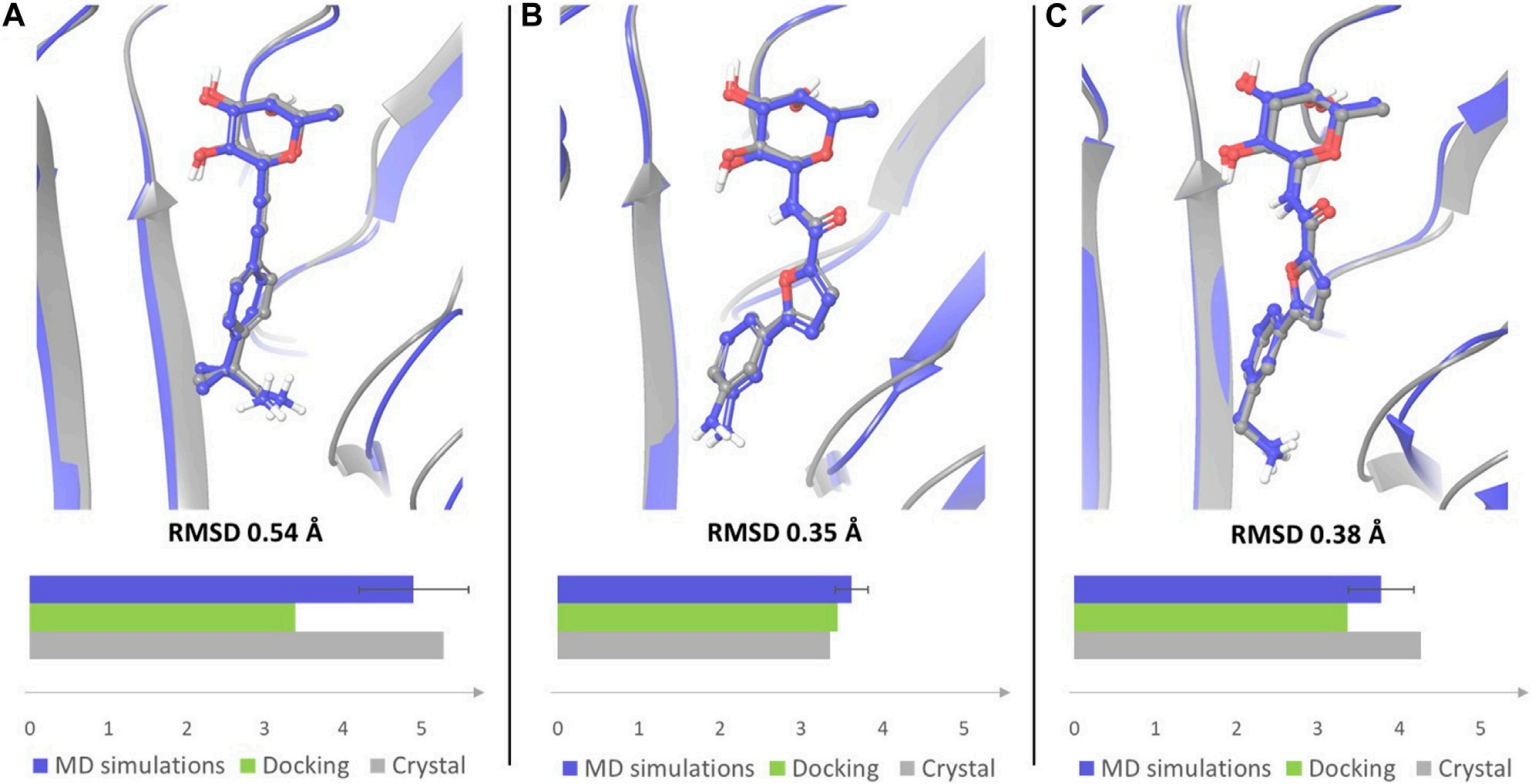
Overlay of the crystal complex of compound 1 **(A)**, 3 **(B)** and 4 **(C)** (gray) with a frame from MD simulations (blue). Frames from the MD simulations were aligned with the crystal structures by protein binding site alignment, then RMSD values were calculated on the heavy atoms of ligand in-place. The depicted frames were selected among those with the lowest RMSD values. For each ligand the distance between the nitrogen atom of the terminal amino group and the carbon atom of Asp70 carboxylic group was monitored in the crystal structure of the complex, in the docking pose that best fits the experimental binding mode, and in MD simulations. The results are displayed in the bar chart (distance in Å). The average distance with error bar calculated over two simulations for each ligand, is reported for MD simulations.

A similar trend could be assessed when monitoring the presence of a salt bridge between the terminal amino group and Asp70 side chain, which was evaluated by measuring the distance between the nitrogen atom of the amino group and the carbon atom of Asp70 carboxylic moiety (Supplementary Figure S4). Comparing the average MD values with those observed in X-ray structures and in docking poses revealed that the average distance calculated by MD simulations for ligands 1 and 4 is closer to the value observed in the crystal structures than the distance measured in docking poses (Figure 8). The absence of a water solvation model in the docking calculations tends to enforce a charge-charge interaction between the amino group of the ligand and the side chain of Asp70 (see Figure 2A for 1 and Figure 2D for 4), which is dampened by the explicit water model employed in MD simulations. Therefore, MD data provide a better agreement with X-ray structures in which the terminal amine of the aglycone part is actually involved in water mediated interactions with this residue. Ligand 3, which is shorter in length and less flexible than all other examined compounds, maintains a similar binding mode in all the available computational and experimental structures, displaying the lowest values of the distance between the terminal nitrogen atom and the carbon atom of Asp70 carboxylic group (3.35 Å in the crystal structure).

Finally, MM-GBSA free energy calculations on frames selected along the MD trajectories provided roughly comparable averaged binding energies for all complexes and thus did not allow to discriminate against the weakest ligand in this group, compound 2. Despite comparing similar ligands binding to the same protein, approximations inherent in the method might introduce significant uncertainty in the results, confirming that the reliable estimation of the binding free energy remains one of the most challenging factors in the study of carbohydrate-protein interactions (Hadden et al., 2015).

## 4 Conclusion

Targeting the N-terminal domain of BC2L-C lectin with glycomimetic antagonists holds promises for anti-adhesion and anti-biofilm therapy against *B. cenocepacia*, a globally spread multidrug-resistant bacterium which is associated with fatal pulmonary infections of cystic fibrosis patients. To develop a computational workflow for the design of these antagonists, we evaluated the use of molecular docking in the protein trimer, followed by refinement using MM-GBSA re-scoring and MD simulations in explicit water. Computational results were compared to experimental data derived from X-ray crystallography and to ITC affinity determination. The five glycomimetic bifunctional ligands 1-5 that we recently reported were selected for this study. The bifunctional ligands, which include β-fucosyl alkynes, β-fucosyl and β-L-galactosyl amides, can simultaneously bind the sugar binding site and a vicinal region at the interface of two monomers. In docking calculations (Glide XP) the fucose or fucose-like anchor of each ligand fully fits the binding mode observed in available crystal structures and the aglycone portion establishes stabilizing interactions with lectin residues in the vicinal site that globally fit the experimental (X-ray) observations. T-shaped π-interactions between Tyr58 and the ligands’ aromatic moiety, that are visible in all available crystal structures, are well described by the docking protocol. Ionic/polar interactions between Asp70 and the terminal amino group of the ligands are also in global agreement with the available crystal structures, but somewhat overestimated by the docking calculations, especially the salt bridge. Conversely, van der Waals interactions between the protein and the methyl groups of the ligand that are experimentally observed in the X-ray structure of the BC2L-C-Nt/1 complex are not fully appreciated by the docking scoring function. These results are representative of the difficulties of scoring functions in reproducing the energetics of sugar/lectin complexes, that are formed at binding sites largely solvent exposed. To improve the description, MM-GBSA free energy calculations were adopted to re-score the docking poses. The results basically confirmed the ranking provided by Glide XP for all ligands, except for 1, where MM-GBSA appears to recognize the hydrophobic complementarity between the methyl groups on the benzylic carbon and the underlying hydrophobic cleft of the protein as a stabilizing feature of the glycomimetic/BC2L-C-Nt complex. This re-ranks the poses and identifies the X-ray binding mode as the lowest-energy one.

MD simulations in explicit TIP3P water of all five complexes were run for 2 × 500 ns, starting from the two lowest-energy poses selected based on MM-GBSA rescoring. An overall stability of the complexes emerged from the analysis of the trajectories, suggesting that the binding site at the interface of two BC2L-C-Nt monomers is somewhat pre-organized to host the bifunctional ligands. Small variations of the RMSD values calculated on the protein backbone are observed in all simulations, supporting the lack of large conformational changes in the trimer. Similarly, low fluctuations of the RMSF values for residues at the binding interface proved the stability of the monomer interface involved in ligand binding during the simulations. For the ligands, both the RMSD values calculated on the sugar anchor (heavy atoms) and examination of the key interactions of this moiety with the protein show that the sugar maintains the starting conformation and all important interactions with the protein throughout the simulations, including direct and water-mediated hydrogen bonds. This last observation, combined with analysis of water occupancy around the ligand, points out that during the simulations TIP3P water molecules occupy similar regions as the two water molecules conserved in crystal complexes and retained in the docking studies as w1 and w2, establishing the same interaction network.

The aglycone portion showed larger mobility in the simulations, as in most ligands it contains a flexible side chain that can rotate and adopt different conformations. The T-shaped π-π stacking interactions between the aromatic ring of the ligands and the side chain of Tyr58 are conserved throughout the simulations, although a modulation is observed depending on the nature of the fragment-derived portion (see Figure 7). Similarly, the methyl groups of 1 establish van der Waals contacts in the underlying protein cleft during more than 50% of the simulation (see movie in Supplementary Material). For all ligands, the side chain of Asp70 is the main partner for the polar interactions formed by the terminal amino group. MD results match better than docking poses the experimental X-ray structures, in which the terminal amino group of the ligands is involved in water mediated interactions with Asp 70. Indeed, the average distance calculated by MD simulations for ligands 1 and 4 is closer to the value observed in the crystal structures than the distance measured in docking poses. The overestimation of charge-charge interactions forced by the absence of a water solvation model in docking calculations is likely relieved by the explicit water model employed in MD simulations. Moreover, the improved description of polar interactions provided by MD simulations in explicit water appears to detect an increased mobility within the binding site of ligand 2, which allows its terminal amino group to fluctuate between Asp70 and Ser119. This could lead to an unstable interaction with the target protein and likely represent a discriminating trait of the weakest ligand of the group. The computational workflow here reported proved capable of providing a reliable description of the interactions between glycomimetic bifunctional ligands and BC2L-C-Nt, thus qualifying as a suitable protocol for the structure-based design of improved BC2L-C-Nt ligands as novel antimicrobials with antiadhesive properties.

## Data Availability

The raw data supporting the conclusion of this article will be made available by the authors, without undue reservation.
