# Supplementary material for: Glycomimetic antagonists of BC2L-C lectin: insights from molecular dynamics simulations
Source: Front Mol Biosci. 2023 May 31;10:1201630. doi: 10.3389/fmolb.2023.1201630 (PMC10264699; doi:10.3389/fmolb.2023.1201630)
Supplement: Supplementary file 2 [file Table1.DOCX]

Supplementary Material

Plots of RMSD values for the sugar moieties 2

Fucose interactions analysis from MD simulations 3

Plots of RMSD values for linker and fragment moieties 4

Salt-bridge interaction analysis from MD simulations 5


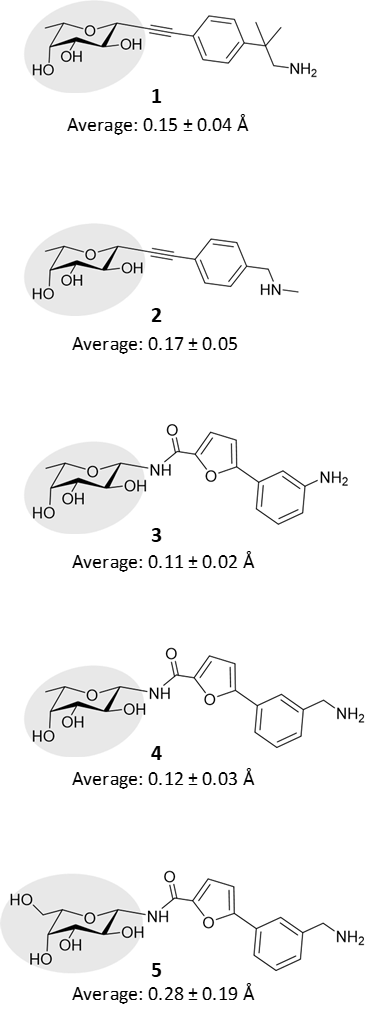

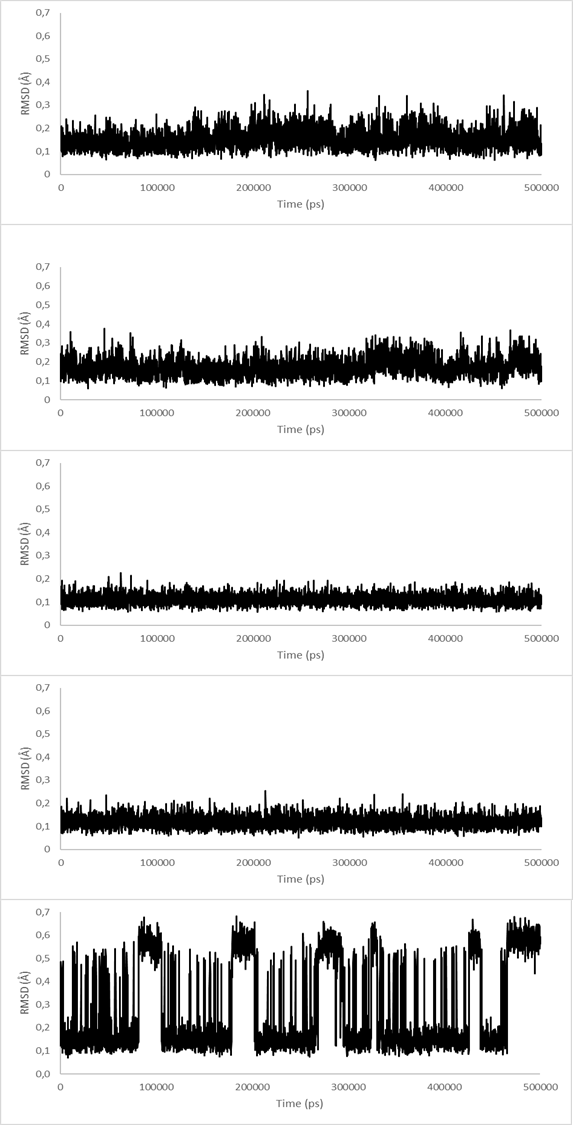


Figure S1. Plot of the RMSD values calculated on fucose or galactose moiety. Superimposition was done on ligand heavy atoms and then the RMSD values was calculated considering only the atoms of the sugar part using the first frame as a reference. No remarkable variations in RMSD values occur during the simulations for the ligands containing fucose, while larger fluctuations are observed for ligand **5** containing the galactose. In the latter case, the rotation of the hydroxyl group in position 6 allows the sugar to adopt different conformations. Only the plot obtained from one MD simulation is shown for each ligand, as similar results are provided by the second simulation run.


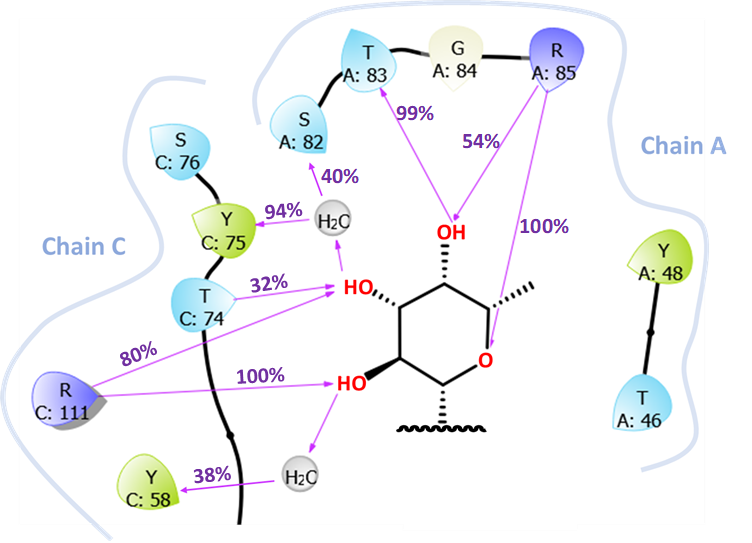


Figure S2. Summary of interactions established by the fucose moiety in bifunctional ligands with adjacent BC2L-C-Nt residues. Every hydroxyl group can form several H-bonds, including water-mediated H-bonds, with different protein residues. The oxygen atom of the sugar ring interacts only with Arg85 from chain A. The percentages of H-bonds have been calculated for each MD simulation considering the 5000 structures saved. Here, the average values assessed over all MD simulations (eight simulations, each 500 ns long) of ligands **1**, **2**, **3** and **4** are reported for each interaction. The following geometric criteria for protein-ligand H-bond have been applied: distance between donor H atom and acceptor atoms ≤ 2.5 Å (D—H···A); a donor angle of ≥ 120° between the donor-hydrogen-acceptor atoms (D—H···A), and an acceptor angle of ≥ 90° between the hydrogen-acceptor-bonded_atom atoms (H···A—X).


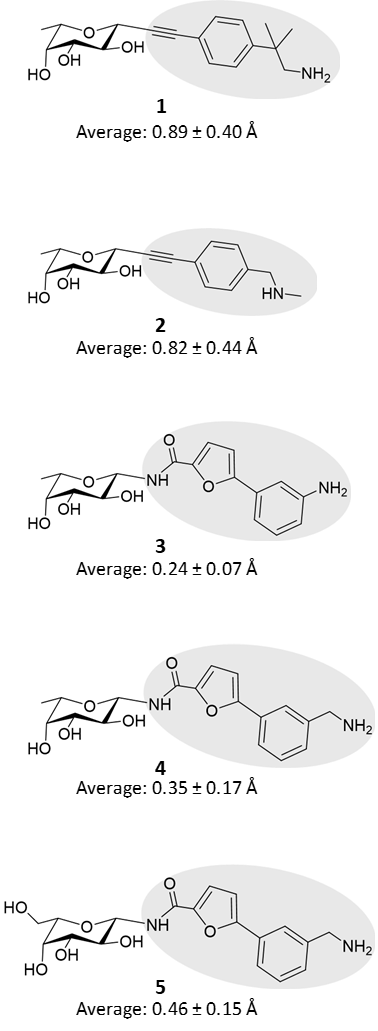

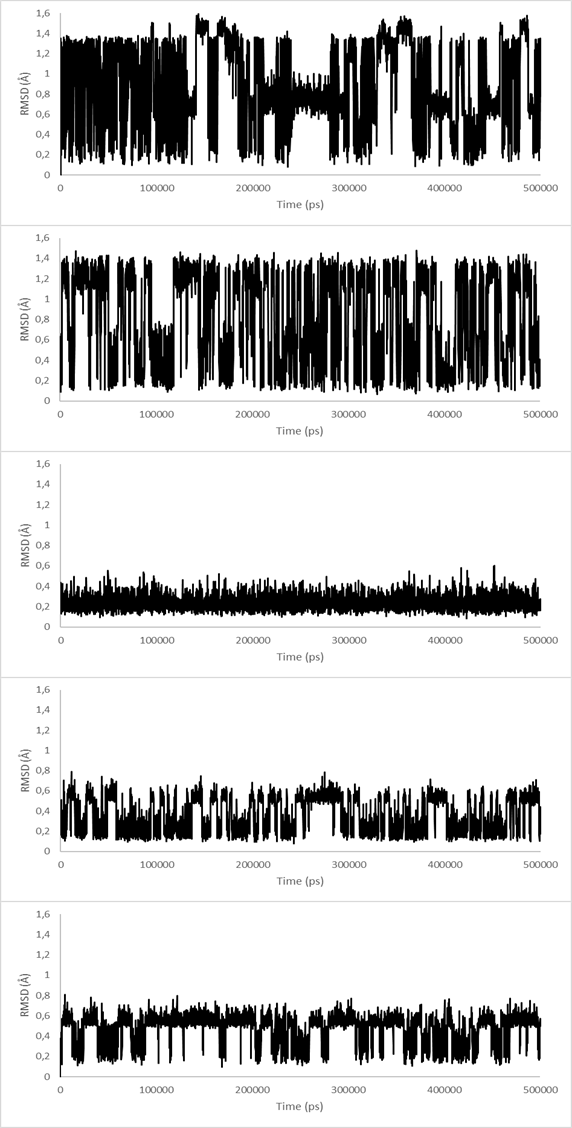


Figure S3. Plot of the RMSD values calculated on linker and fragment moieties. Superimposition was done on ligand heavy atoms and then, the RMSD values was calculated considering only the linker and the fragment part using the first frame as a reference. Fluctuations of RMSD values are observed mostly for fucosylalkynes **1** and **2**, due to the presence of flexible side chains. Small variations of RMSD values are noted for amide ligands **3**, **4** and **5**, which comprise an amide linked to two connected (furan and benzene) aromatic rings. In particular, compound **3** shows the lowest oscillation of RMSD values among the 5 ligands, as a result of the lack of rotatable bonds.

Figure S4. Percentage of salt bridge between the terminal amino group of ligands and Asp70 side chain calculated considering the structures saved from each MD simulations. Average values assessed over two MD simulations of each ligand are shown. The same trend was observed monitoring the hydrogen bonds between the Asp70 carboxyl group and the ligand amino group. Compounds **4** and **5** are more extended in the binding site toward the aspartic acid compared to ligands **1** and **2** thus, they are more engaged in the salt bridge formation. The presence of the salt bridge was evaluated monitoring the distance between the nitrogen atom of ligand’s amino group and Asp70 carbon of COO- group, and considering the interaction as properly established if this distance is ≤ 4.0 Å.
